# Supplementary figures and images for: Bioecology of fall armyworm Spodoptera frugiperda (J. E. Smith), its management and potential patterns of seasonal spread in Africa
Source: PLoS One. 2021 Jun 11;16(6):e0249042. doi: 10.1371/journal.pone.0249042 (PMC8195398; doi:10.1371/journal.pone.0249042)

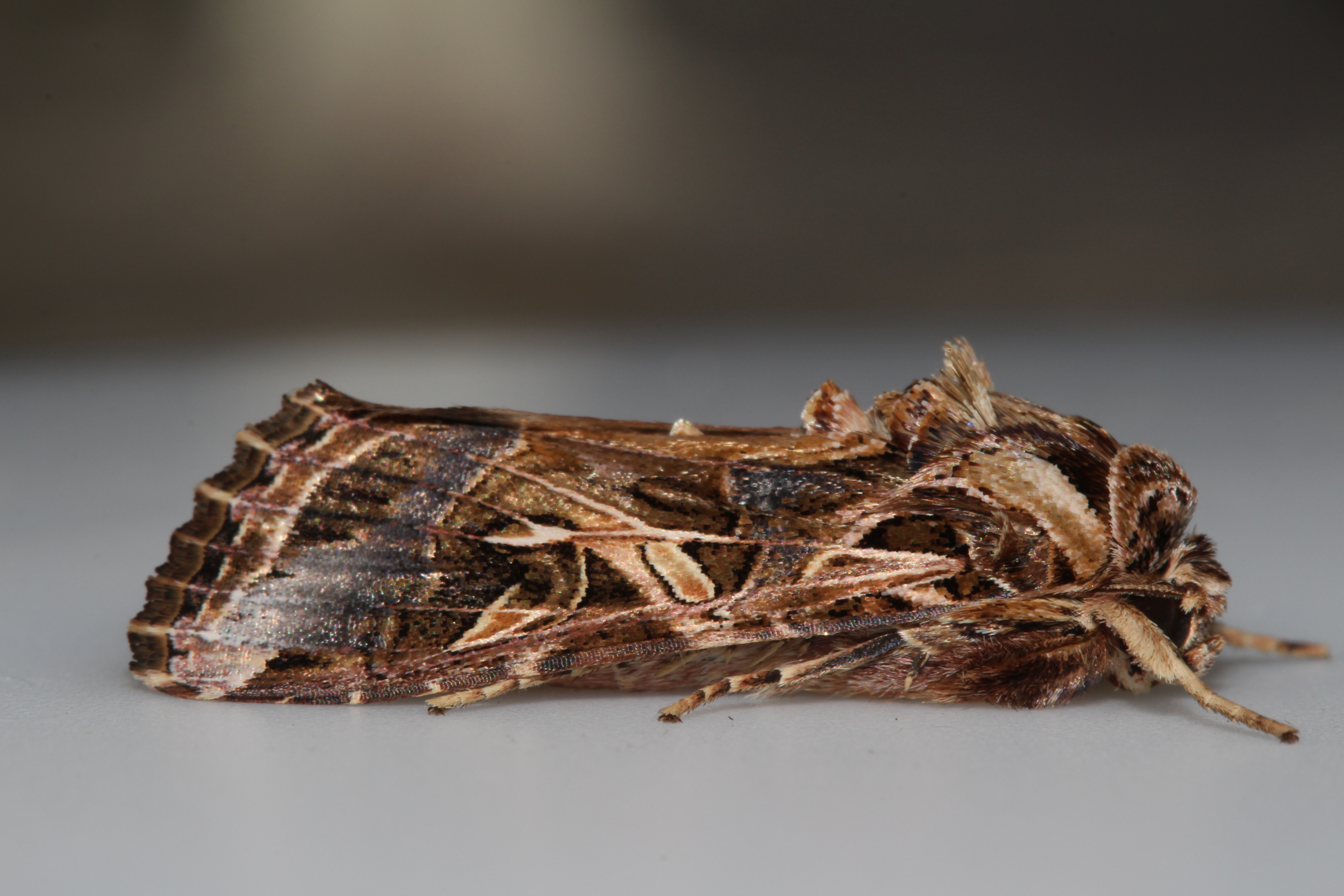

Supplement: S1 Fig — (JPG) [file pone.0249042.s001.JPG]
