# Supplementary material for: Bioecology of fall armyworm Spodoptera frugiperda (J. E. Smith), its management and potential patterns of seasonal spread in Africa
Source: PLoS One. 2021 Jun 11;16(6):e0249042. doi: 10.1371/journal.pone.0249042 (PMC8195398; doi:10.1371/journal.pone.0249042)
Supplement: Attachment — Submitted filename: Responses to reviewers_PLOS I_02-10-2020.docx [file pone.0249042.s002.docx]

| Comment | Response |
| --- | --- |
| In this paper, the authors examine a wealth of data gathered by the CBFAMFEW monitoring  system in Africa, especially extensive data on fall armyworm (FAW) adult and larval populations across 6 countries in eastern Africa. | Thanks for the encouraging comment |
| The FAW is a devastating pest of many crops,  including especially maize, that was recently introduced to Africa from the Americas and it has spread rapidly across the continent and into Asia. I agree with the authors that the CBFAMFEW system has a valuable role to play in understanding FAW dynamics in Africa, and should continue to be supported. There are valuable data summarized in this paper, and the authors have the laudable goal of using them to gain ecological insights that can help development of practical pest management strategies. But there are some significant shortcomings with how the authors attempt to analyze, present, and draw conclusions from these data. | We thank the reviewers for taking their time to review the paper and point out the major shortcoming which we have tried to address in this point by point response. |
| There is a problem with how the authors are relying on correlations of survey data on cropping systems, rotations, chemical vs. bio pesticides, rainfall, etc. gathered from regional and continent-wide surveys to infer regional and continent-wide effects on FAW populations as measured by scouting and pheromone trap captures. | We thank the reviewer for bringing out the major shortcomings in terms of data analysis and inference. We have to that effect consulted with our data experts of which some are co-authors of this present paper to improve the data analysis for appropriate interpretation. As such new analysis have been presented and statement have been adjusted accordingly. |
| Much of the paper is based on the idea that levels of infestation and FAW densities are "influenced" by or "affected" by these variables. However, such conclusions cannot be drawn in the way the authors have done, because they are based on correlative approaches where cause and effect are unclear. | We thanks the reviewer for the comment. The paper is largely to explore the bio-ecology of FAW to get insights on FAW dynamics and FAW behavior in its new home. The paper is providing preliminary information considering the variability of maize ecologies and farming systems to explain how FAW both adult and larvae are thriving which has implication on sustainable management technologies. Therefore, we have tried to relate the recorded data with what is available in literature. |
| In addition, for most cases where the authors claim a cause and effect relationship, the data on FAW for the different independent variables are not shown.  So, for example, the authors show the percentage use of different classes of pesticides used in different countries in Fig. 6, and then mention whether the larval counts or adult counts of FAW were high or low. We do not see the insect data, we are just given impressions of cause and effect of pesticides on vague, undefined categorizations of pest density by the authors. | We thank the reviewer once again for this pertinent comment. I would like to mention that data on FAW density both adult and larvae in a certain location are simultaneously captured along the type of chemical or biopesticide use and quantity used. This type of data is only indicative of farmers’ response to FAW infestation in various countries. Though this was not the focus of the paper, we do agree that more required in establishing the cause and effect relationship of pesticide use and larval/adult counts. |
| The survey data are simply that: survey data. If the authors think there may be a cause and effect pattern between some of these variables and FAW populations, they must test it statistically by comparing means of different variables, and these tests should be broken down at least by region. A significant correlation coefficient is not sufficient to declare an effect or influence of a variable on FAW populations. | We have re-visited the data analysis interpretation with help of Biometrician and hence the statistical statements have been modified and presented appropriately as suggested. |
| The most promising data in this study in terms of providing useful insight into FAW dynamics is the relationship between pest density and phenology of the maize crop (not other crops as implied in the current version - this study should be all about maize for which there are the most data). I think the authors have the data to address this. My suggestion is to focus the paper on this aspect and analyze it thoroughly and appropriately, looking at different regions separately as well as together. | The review may have a point however; in the overall strategy to control FAW it is important to consider East Africa as a region and Africa as continent. Aspect such as variability of season, cultural practices and even staple cropping become very important variables in the entire Matrix to control FAW. Moreover, additional data from other sources e.g. rainfall, temperature, wind direction, land cover, irrigation and type of vegetation etc play an important role in pest development. This study provides a modest contribution in the insight on FAW bioecology and there is a lot more to tell beyond the relationship between pest density and phenology of the maize crop.  Furthermore, African cropping system is rarely monocrop and hence to consider maize only would be underutilizing the recorded data which considers all companion crops grown. |
| In addition, the heat maps of infestation intensity over time is valuable and could be presented, but the authors can only speculate about the cause of changes over time being movement out of hot spots. Such possible patterns may be caused by moth movement, but this must be tested in some other way than simply looking at the heat maps and concluding "migration" is the cause. The other data are valuable as a survey of agronomic practices in different parts of the continent, which may impact FAW populations and their management; however, the type of data collected in this regard can only be used to generate hypotheses for testing possible cause and effect relationships experimentally in future studies. | We thank the reviewer for his brilliant remark of which we take good note. We presented heatmaps on quarterly basis consecutively and describe the dynamics of FAW in the region.  Unlike many other approaches used in the prediction of pests, we have rich data (abundance) which have been collected directly on the ground. Therefore, the heat maps are generated from these data of abundance. Spatial analysis beyond heatmaps gave poor predicted values and hence results were abandoned, remaining with heatmaps only. Much as we agree with the reviewer’s concerns on spatial statistics, we should not underestimate the migratory capabilities of adult FAW and the realities on the ground (e.g. irrigation farming in those hotspots) which underpins the suitability of FAW.  Therefore, this study a preliminary analysis that generates invaluable information on the understanding of FAW dynamics. More analytical modelling studies are underway |
| Other comments below: |  |
| Throughout, the authors should talk about larval and adult numbers (counts, captures, etc.) rather than "scouting" or "trapping" numbers. | Done |
| L57: change "constitutes" to "adds" | Done Line 59 |
| L81: I think the authors must mean the onset of Spring, not Fall. | Done Line 89 |
| L97: change to "...protocol could be valuable in improving our understanding of FAW..." | Done Line 100 |
| L110: delete first "countries" | Done Line 114 |
| L113: should "Community Focal Points" be "Community Focal Persons" as in L160? "Persons" would make more sense. | Done Line 117 |
| L123: change to "and early warning" | Done Line 127 |
| L124-125: change to "To coordinate data collection and transmission, mobile phones were loaded with..." | Done Line 128-129 |
| L126: delete "was provided to contributed to the national capacity that enabled the data to be  generated" | Done |
| L127-128: change to "The FAMEWS app is available in Google Play in 13 languages and was downloaded by farmers and extension workers." | Done Line 129-131 |
| L131: delete "this" | Done |
| L134: change to "density across a wider area of SSA" | Done Line 136-137 |
| L136: delete "Setting up of" | Done |
| L137: change "of pheromones" to "baited with FAW pheromone". | Done Line 141 |
| Authors need to indicate the source of the pheromone (i.e., manufacturer) | Done Line 143 |
| L138: change "were placed in the field" to "were deployed"; also change "counting" to  "monitoring" | Done Line 141 |
| L140: not sure what the authors mean by "bush". Do they mean an actual bush (a single plant)? Or do they mean "brush" in the sense of a nearby uncultivated brushy area? Suggest changing to  "...and a second in nearby brush" | Actually, the term “bush” refers to outside the maize fields and the concessions whereby alternative FAW hostplants are likely to be found e.g. other grasses etc. We have changed it to “outside the maize field”. Line 146 |
| L142: change "every week" to "weekly" | Done Line 148 |
| L142-143: pheromones should be specific to the species they attract. However, sometimes cross attraction with another species can be a problem. To what extent did the pheromones in this study attract other species to the trap? Was it a problem? | Thanks for the comment. Yes, you are right the pheromone traps could attract another species, but in our case, we followed a protocol of sorting trapped adult. Moths are grouped in term of species and the different groups are recorded. The issue of specificity of traps was often reported however, in this case a clear distinction was made between FAW and other moths attracted through the various training and sharing of FAW identification manuals. |
| Was identification of moths difficult or  impossible due to losing wing scales during several days of being in the trap? | Thanks for comment. No/yes, the identification of the moths was not difficult as the CBFAMFEW programme catered for a comprehensive training on FAW lifecycle and FAW identification at adult and larval stage. This is indicated in the M&M section “Further training on regular field scouting and detecting the pest at various stages was also provided to the CFPs.” |
| Were moths killed by an insecticide strip or something when they entered the trap, or were they simply held without immediate killing? | Yes indeed, the monitoring kit was composed of a yellow trap bucket trap and pheromone sachet provided by Russell IPM. The kit also contained a toxicant which immobilized the moths once attracted to the device. Line 142-145 |
| Also, bucket traps can fill quickly if a species is abundant. How often did a pheromone trap fill up during a week of captures? (This would be exacerbated if large numbers of a non-target species was also being trapped.) If it fills up, or near the top, the numbers of FAW in the trap will be an underestimate of the numbers it would have trapped during the week if bucket capacity was not limiting. I realize it might not have been possible under local conditions to check the traps more often than weekly, and this does not negate the importance of the data, but the authors need to discuss any issues like this because it can affect the interpretation of the data. | We thank the reviewer for his pertinent comments. Although data was collected weekly, scouts were conducted twice a week. This enabled the extension officers and community for persons CFPs to monitor traps in case they get fill-up. The situations whereby traps were filled up to an extent we could not record data accurately were rare, but the frequency of visit enabled agents to clean and reset the traps. |
| L144: delete "On the other hand,”; Also, change "right from" to "beginning at" | Done |
| L147: change "farms" to "fields" | Done Line 152 |
| L148: delete "random different" | Done |
| L149: change "other" to "non-target"; Also, change to "...counts from pheromone traps, and  field scouting data..." | Done Line 154 |
| L150: what is meant by "percentages of FAW infestation"? Do the authors mean "percentage of plants infested with FAW"? Or "percentage of plots with an FAW infestation"? Or something else? | Thanks for the meticulous comment. We meant the mean percentage of plants infested. |
| L173-174: a dependent variable is plotted against the independent variable. So should say FAW density was plotted against the rainfall data. | Another meticulous comment, indeed. The rainfall data were sourced on a monthly basis in millimeters. We have corrected this everywhere in the manuscript. |
| L173: What kind of rainfall data? Total cm per year? per day? per season? Mean rainfall per  some unit of time? In other words, what units of rainfall were used in the analyses? | Done- The rainfall data was sourced on a monthly basis in millimeters. |
| L175-178: Did the authors identify "downpours" and then compare downpours to pest abundance? If so this needs to be described. If not, perhaps the authors mean to say that "We interpreted effects of rainfall from a pest management perspective...", instead of "used rainfall data"? | Done Line 183-186 |
| L177: change to "negatively affect" | We have revised the statement |
| L177: Rainfall can both negatively and positively affect "general plant health". Both too much or too little rainfall can negatively affect plant health. | Revised in L185-186” Rainfall can both negatively and positively affect the general plant health, particularly in rain-fed cropping systems” Line 185-186 |
| L179-180: Change to "based on both trap and scouting data" | Done Line 187-188 |
| L182-184: It is unclear what the authors mean. What is meant by "points in a location"? "points” of what? fields with FAW present? sample points within a field with FAW? What is a "cluster" and how is that defined? Or do the authors mean that the more "points" of FAW infestation within an area of sampling, the higher the value in the algorithm generating the heat map? If the latter, or something similar, was the method for creating the heat map, how did the authors account for differences in intensity of sampling in different areas? For example, if FAW density was the same in two 10km^2 areas, but the first area had twice as much sampling as the second  area, would the first area appear hotter on a heat map than the second? | Addressed. We clarified on the meaning of point and also explained how the kernel density tool works. L192-194 |
| L183: change "significant" to "substantial"; in a scientific paper "significant" should only be  used when describing results of a statistical test. | Done |
| L188: change "follow" to "fallow"; Also, what is meant by "main and second seasons"? Are there two seasons per year in northeastern Africa for growing maize? | "follow" changed to "fallow" Line 201.  In the six target countries of CBFAMFEW there are two raining seasons.  <http://www.fao.org/giews/countrybrief/country.jsp?lang=en&code=KEN> |
| L193: change "In specific" to "Specifically" | Done |
| L191-194: The authors state "we overlaid the quarter FAW density heat maps in 2018 and 2019 to indicate the movement of the adult moth across the region." This strategy cannot by itself "determine FAW migratory patterns". The only thing the heat maps portray over time is the spatial patterns of change in FAW abundance in different areas, they cannot indicate the cause of those changes. Changes in FAW density could be caused by migration, by local reproduction and mortality (of adults or larvae), or some combination of both. How do the authors know changes in density on the heat maps were caused by migration?  They must have independent evidence, or previously established specific knowledge, that FAW migration is occurring during those times to and from those places (e.g., radar data, sudden increases in trap captures associated with winds, etc.) before they can make such a claim. The most the authors can do along this line is hypothesize that some of the changes in the heat maps reflect migratory movement (explaining why they think this could be happening), and that this hypothesis will have to be tested with further research. | We thank the reviewer for raising this point, and we took a kind note of it. We are indeed aware of the information provided and agree that our current heat maps could only be used to hypothesize the FAW migratory pattern. In this revised manuscript we have rephrased our results and discussion to address this comment.  The CBFAMFEW approach is the first of its kind to collate data in short interval period over a long period of time to portray FAW dynamic in Africa. It is therefore a valuable asset to describe the special patterns of change in FAW and explain what caused this change based on knowledge of FAW bioecology and previous information in other regions as well as reality on the ground. We indeed do have evidence on this in the Americas and other ongoing initiative of USAID and the FAO on the continent.  We agree that additional data will be required to strengthen the idea of movement. We indeed attempted to integrate rainfall data but is needed in future as explained earlier. This study advocates mainly for the expansion of CBFAMFEW approach for more accurate data. |
| L195: Why "intersect" sites? This word doesn't seem relevant to the phenomenon it is describing. | We thank again the reviewer. We use the intersect technique (GIS) to distinguish the different quarters of FAW expansion on the heatmaps. This is a technique employed to also describe potential hotspots (and recessions) and expansion areas over the quarters. We will be happy if reviewer can suggest a different wording. |
| L199: "FAW counts and FAW adults in traps"; What is the difference? Counts of what? Of larvae during scouting? The terminology is imprecise so the reader cannot know what is being referred to. | Thanks, we have corrected this throughout the text. Trap catches are now referred to as adult counts and scout counts are now referred to as larval counts |
| L209: This is a difficult sentence to follow. "we assumed that": was this assumed or is this what was being tested? | The sentence has been revised |
| Tables 1,2,3: The tables show only values related to statistical tests. These are of some interest but belong in supplemental material. Instead, the authors need tables that show the actual data. For example, the mean trap counts and scouting counts for each crop, perhaps by district or some other geographical subdivision. | We thank the reviewers once more. We have reanalyzed the data with improved outputs and interpretation. We have opted to display the regression estimates, their significant levels as well as the incident rate ratios (IRR) which are an indicator of relative risk compared to a chosen reference level of factor. IRR are an adequate presentation for counts data - IRR is congruent to Odds ratios in binary data. |
| L227-228: Maybe I missed it, but somewhere in the methods the authors need to explain what is meant by "push-pull technology" and "seasonal cropping system" | Done |
| L232-234: show the data, not just results of analyses | This has been revised. |
| L238-242: show the data, not just results of analyses | This has been revised |
| L245-249: show the data | This has been revised |
| L288-291: cannot say these are weak correlations, inverse or otherwise. With p-values of 0.5-0.9, can only say there was no correlation. Same for Lines 293-294, 297-298, 300-302, 303-306. If p>0.05 there simply is no correlation, period. | This has been revised |
| Fig. 8: It is unclear what these data are. I think the authors must be showing numbers of FAW per crop phenology stage totaled across all sample sites in the 6 countries of eastern Africa. What is meant by "Number of plants" on the other y-axis? Total number of plants infested with FAW? Total number of plants per phenological stage? Or something else? The data are potentially valuable, but the authors should break this down by region and give the insect data as means +/- SE, then test for differences in means between phenological stages within those regions. As is, there is no indication, or taking account of, variation in populations between regions, which could be high. | Thanks for the constructive comment. Legend has been corrected and we have provided a new table which shows adult and larval counts per crop stage and across countries. |
| L323: show the data by country | Done |
| L440-442: the authors cannot say there were correlations when the p-values were >0.05. | This has been corrected |
| L492-495: the data in this paper are consistent with this interpretation (spread from hotspots of infestation), but they do not "demonstrate" it, for the reasons explained earlier. | We thank the reviewer once more CBFAMFEW additional studies are underway to confirm the spatial pattern change. Independent evidence, or previously established specific knowledge, that FAW migration is occurring during those times to and from those places (e.g., radar data, sudden increases in trap captures associated with winds, etc.) has been demonstrated in the Americas. Also, there are crucial elements of FAW physiology (migratory capability and absence of diapause) which need to be considered in the interpretation of our results.  We appreciate that the reviewer agrees that the data support our interpretation of the results and we have provided explanations. |
| Reviewer #1: |  |
| This manuscript reports on a remarkable dataset from six countries, with five maize growing regions in each country, and 10 villages in each district over two years. As such, this represents a huge set of 600 individual data points. The topic is very important and good data are needed on this damaging species. However, it is not acceptable for publication in its current form, largely due to questions surrounding the methodology. | Thank you for the encouraging comment. |
| My biggest issue is that the statistical analyses are not well described. As I read it, models were constructed to see what variables were important to predict FAW counts in the fields and in traps. The authors state that they used AIC for model selection. While it is good to select models with low AIC values, this should be used to see that model assumptions, such as linearity, normality, etc. are being met and not to include or eliminate variables. A more common technique is to create a full model and then to test reduced models using backward elimination of non-significant terms at 0.05 level. | We have revised the statistics and provided clearer explanation of the methods and models used with the help of data experts and a Biometrician who are now co-authors of this paper. |
| In addition, these data are inherently spatial, but linear models were used. Were any tests done to see if there was spatial autocorrelation? If there is, the model should structure the covariance in such a way as to take this into account. Furthermore, it wasn’t clear what base packages were used in R for the analyses. | The farmers’s field under observation are a random sample and hence are the traps placed in the field. The analysis of data is at field level – thus our unit of observation. Though the data are collected in a spatial sense, the variability in the FAW abundance can still be ascribed to the various factors in play in the fields namely; the main crop under cultivation, cropping system, crop rotation whose effect on FAW abundance/counts have been examined separately using generalized linear model that assume a negative binomial distribution error to cater for overdispersion in the FAW counts. This model uses the MASS package which has been included in the ‘Data analysis section’. |
| I also am unclear exactly how the correlation analyses were done based on the description in the methods. Moreover, looking at the results, inferences are drawn on “weak” relationships for P values as low as 0.9 (line 291). A typical P value for a statistically valid result may be around the 0.1 or 0.05 level to avoid false positives. | Thank you for the constructive comment. We have revised the statements that caused the confusion. We do thank the reviewer once again specifically for this comment. |
| Finally, the chi-squared analyses were not well described. I’m still wondering what “we assumed that the frequency distribution of FAW in the six CBFAMFEW countries observed is consistent with the categorical variables” means (lines 209-210). Reported degrees of freedom in line 312 are 340 and 248. Do those represent analyses for two years (roughly equal to 600 sampling points)? | The section has been revised with assistance from Biometrician. Some analysis have been eliminated as they were a repeat of what was already analyzed using glm. |
| I had numerous smaller concerns as well. For example, the reader doesn’t know what lure was used. This is important since different strains respond differently to pheromones in different geographical locations (Unbehend et al. 2014- Geographic Variation in Sexual Attraction of Spodoptera frugiperda Corn- and Rice-Strain Males to Pheromone Lures). | Thanks for the comment. Monitoring kit (including lures and traps) were detailed in the revised manuscript. Monitoring kit (including lures and traps) were purchased from Russell IPM. Contacts of supplier have been added and this has been added in the Materials and Methods section |
| Furthermore, scouting is described as being done by “farmers and extension workers”. How many people scouted? Was any sort of quality control done (like following a selected portion around to be sure the findings were correct)? There is mention of a national coordinator to validate data (line 159), but no description of the validation. | Thanks for the comment. Scouting was done by both extension officers and community focal persons who were also trained and given a mobile device and traps to collect data. The quality control was done though the trainings as we mentioned to insure the correctness of the findings |
| How was African armyworm damage distinguished from FAW damage? In my experience, you cannot distinguish noctuid species simply based on damage in whorl-stage corn. How many larvae were identified as a check? | Thank you for the meticulous comment. Yes, you are right. But during CBFAMFEW piloting, a module training was conducted on FAW identification and how it differs from African armyworm. Training module can be access on this link: <https://www.cabi.org/isc/FullTextPDF/2019/20197200157.pdf> |
| Reviewer #2 |  |
| It is extremely important that the authors pay attention to detail in this paper. The language they use is poor, which leaves a lot open to interpretation. It is not possible that all the authors read and approved this manuscript. | Thank you for the comment. Most of the comments have been addressed as suggested by the reviewers. The paper was edited by a professional language editor and co-authors have significantly provided substantial editing. Statistical analysis were re-visited by a Biometrician as suggested. |
| The introduction needs restructuring. A lot of the text on importance of crops and different constraints are unnecessary. Stick to the topic - which is migration.  The study does not address the ‘’Bioecology of FAW’’, or Management. It is a survey of cropping systems and abundance of the pest in different regions. How does migrations patterns actually go? They do not answer that. How does it compare relative to those in North America? | We thank the review for the remark. Though the topic of the paper is not exclusively about migration, we have added a statement to cater for FAW migratory pattern in the US and the need for understanding the scenario in Africa, a different environment (Line 82-85). The paper discussed the importance of Community-Based Monitoring Forecasting and Early Warning in improving the understanding of FAW bioecology. Since this a component which is often overlooked in Africa, the introduction motivated the importance of this approach and its complementarity with current FAW control strategies. |
| The discussion is superficial and no mention and discussion (as far as I could ascertain) of the extremely important work of Baudron et al. is made, although they do refer to cropping systems in this study in East Africa. | We thank the reviewer for highlighting the importance of Baudron et al. study. In our revised manuscript, we discussed the study of Baudron et al in context of our findings, lines 379, 386, and 398. |
| The abstract contains all that is needed but it is not explicitly accurate. The authors should pay attention to detail, since this is an important paper and important information they want to convey. Throughout the paper they should be accurate in the use of wording when they refer to infestation which is high or low is important. For example, does that mean % infested plants per field or number per plant? | We appreciate the reviewer’s comment on the fact that “*the abstract contains all that is needed but it is not explicitly accurate have revised the definition of percentage infestation*”. Also, we thank the reviewer for reinforcing the importance of our paper and findings. We revised our wording and consistently and accurately explained what we mean by “infestation”. The counting exercise provides details on mean% percentage infested per field. |
| The in-text reference numbers are mostly all wrong. The papers they refer to do not relate to the statements they are referred to as to support. Examples: the references in lines 96 and 101 and corresponding papers in the reference list do not deal with what they are said to. | We apologize for the inconsistencies which are due to content control technicality using a reference manager software. All references have been checked carefully to match the statement. |
| I strongly suggest that the acronym for Community-based Fall Armyworm Monitoring, Forecasting, Early Warning and Management (CBFAMFEW) be used only in the beginning and then some other term is used. | Done |
| The authors should write in the past tense. This is especially the case in the results section and further on. | We thank the reviewer for his suggestion which we take good note. |
| Abstract: Why is this a pilot study? | The study is the first of its kind in the countries where it was implemented given the recent invasion of FAW, hence we thought it is still under piloting phase. Discussions are underway for possible scaling out in other countries. |
| Line 40-41. ‘’sustainable decision making’’ ? this is awkward. Delete. | Done |
| The use of the terminology: scout counts is in correct. Another word would be much more appropriate. This is unscientific. | The terminology has been revised as following: “adult counts” and “larval counts” throughout the manuscript |
| Line 34. This sentence does not make sense. | Sentence has been revised |
| Line 35. biopesticides. Also: please indicate on what parameter of adult populations was there an effect. | Sentence has been revised |
| Line 36. Abundance of ? moths/larvae. Also: ……..with infestation being high. | Data were re-analysed and statement has been changed |
| Lines 46/46. Basic errors in language | Corrected |
| Line 57. Poor language /rephrase. ……Wreaking havoc…….. | Corrected |
| Lines 71-7. Major statements without any references. | Reference has been added |
| Line 82. Rephrase … its residence.. | Done |
| Line 101. This is an inappropriate reference. It refers to sorghum grain quality and not IPM. | Addressed |
| Line 139 . seedling emergence (more than one) | Done |
| Line 150. Please be precise. The incidence (%) of plants per field that show symptoms…. This is confusing since there are two references in this one sentence to infestation (percentages of FAW infestation … and mean plot infestation (plot=field?). | corrected |
| Line 176. Interfere is the wrong word (influence) and ….. what aspect of neonates? | The statement has been revised |
| Line 179. Please define unit area. Does that differ from a ‘location’ in line 183. | addressed Line 184 |
| Line 191. Higher up you described another trap type. Standardize terminology. | Addressed. Standardized to “universal bucket traps” Line 205 |
| Line 215. Where there 4165 target districts? Is that correct? In line 223 it is indicated that there were 5291 districts. | Thanks for the observation and we took a kind note of it. But 4165 and 5291 refer to the number of observations |
| Line 219. What counts are referred to here? please be specific. Line 232. Trap and scout counts…. non-scientific jargon. | The terms trap and scout counts have been changed into adult and larval counts. |
| Line 220. Rephrase sentence | done |
| Line 222. Not clear what is meant by ‘’seasonal’’ in this context. If season and rotation systems were dominant, which other systems are there? | Thanks for the comment. Seasonal refers to maize production mainly driven by rainy season. The FAMEWS App enables to choose the following systems: Push-Pull, Intercropping, Rotation and Seasonal |
| Line 240. Replace ‘’trap counts’’ with the numbers of moths per trap. | Done |
| Line 241. The language and inconsistent use of terminology makes this paper difficult to review. For example: line 241. Scout counts were positively influenced by Cassava and other cereals;>> this means that the whole study was biased. | Once more we thank the reviewers for pointing this out. This part has been rephrased |
| Line 248. This says that Moth counts significantly affected biopesticides. | We took a kind note of the reviewer’s comment. We thought that we have explained this in L259 “*During the surveyed period for the target sites, the quantity of biopesticides used in FAW management was very low (307 liters) as compared with chemical pesticides (10,293 liters)*”. |
| Line 251. A unit are must provide at least for the litres of pesticides used. Volume per number of farms / hectares? | Ok |
| Line 253. Primary? Meaning? | The most commonly used. Rephrased |
| Line 258-259. Which products are referred to here? It reads as if there were different pheromone products. | The statement has been corrected |
| “”Alpha Cypermethrin recorded high counts in Burundi…: this makes no sense. | The statement has been corrected after re-analysing the data |
| Line 313. I assume that trap count here refers to total number of moths in traps (n = 5550). However, it is unclear to what scouting count refers to? | Once more, we thank the reviewer for pointing this out. This has been revised throughout the paper as previously mentioned. Trap counts (adult counts) is the total number of moths in traps. Scouting count refers to the number of infested plants recorded in a sample of 50 plants per field. |
| Line 356. ‘’ other crops are also grown as main crops and play a crucial role on FAW populations’’ What does this mean? This is not explained. | Thanks for the comment. In many countries in East Africa, maize is the staple crop. However, in many instances, maize is planted along with some other equally important crops such as beans, cowpea, cassava etc. We have clarified this in L……………………. |
| Line 368. Relevant host plants. | Changed to alternative host plants |
| Line 396-397. Nothing to do with the study. | Removed |
| Lines 402 – 404. Confusing. Which continent is referred to here. Cotesia icipe is not indigenous to Americas and T. remus not to Africa. | Corrected |
| Line 491. Reference? | Done |
| The manuscript should be restructured. Line 232-234. This section does not fall under the heading 3.1 which indicates: Crop diversity and cropping systems in East Africa, since it deals with infestation data. The same for lines 240-242. | Thanks for the constructive comment. Done |
| The figures are really poor quality and needs tidying up. For example, Y-axes, some indicate % after the number on the axis. Title of Y-axis in some cases: Percentage> But % what?? Did all the authors proof read this manuscript? | These are meticulous observations. We have addressed them all and yes, all authors have proofread the manuscript. |
| Fig. 6A. Y axis. Larvae. Larvae per tree? Per country? Per plant? Y axes have units. | Addressed |
| Fig. 8 not traps count but Trap count of mean numbers of moths. | Addressed |
| Reviewer #3 |  |
| I've attached an marked edited copy. I also had the following comments: |  |
| lines 80 – 81: no, in North America migration starts from Texas and Florida in the late winter or spring (February – May). Populations are noticed in the northern areas in late summer and fall, which is why it is called fall armyworm since an entomologist in Missouri described biology and crop damage | We have amended the section for clarity as suggested by the reviewer |
| line 99: what is the difference between seasonal cropping and rotational cropping? | Thanks for the comment. Seasonal cropping is a farming practice in which the same crop is grown in the same area for a number of growing seasons. In the case of maize, it is mainly rain fed, and the land remain fallow between seasons. While crop rotation is the practice of growing a series of different types of crops in the same area across seasons. |
| lines 137 – 141: what pheromone lures were used, that is, from what companies and what were the components; | This is clarified in L142 of our revised manuscript. Pheromone lures were purchased from Russell IPM, a renown company which supplies lures and traps. |
| why were traps put into two separate areas and was the analysis done for these traps separately; | Thank you for pointing this out. In the CBFAMFEW protocol, two traps were installed per village; one around the crop farm and the other near non-farmland (grassland, bushes, etc). Traps were separated with a minimum of 50 m to avoid any cross interference. The reason for this was to be able to trap adults during and after maize season considering the number of alternative host plants.  But the analysis was not done separately for the two traps. |
| did anyone verify the species of male moths collected – traps in the Americas as well as in Africa attract many nontarget species, several of which look similar to FAW | Yes, as a training was provided to community focal persons (CFPs) on FAW identification. Trap catches were sorted and FAW moths and non-target were grouped separately by the trained CFPs. |
| line 220: cowpeas was not significant for scout counts | This statement has been revised |
| lines 232 – 234: this analysis would be more appropriate if done by country since seasonal cropping is predominate in 4 countries and crop rotation in Burundi and Rwanda | This paragraph has been eliminated as data was re-analysed with assistance of Biometrician as requested. |
| So your results suggest that where you had intercropping you had higher male moths collected and higher numbers of larvae found? | Thanks for the interesting comment. The data has been re-analyzed and this section has been revised. Data show that the risk of catching male adult moth under intercropping is 2 times higher compared to seasonal cropping. However, it is important to note that intercropping is not commonly practiced in the region and it only represented 10% of the data. |
| lines 288 – 307: none of these countries had significant correlations with trap and scout counts and rainfall, so you don’t to mention each country separately. If the significance value is greater than 0.05 (or whatever value you determine is appropriate), then the relationship doesn’t exist – its not weakly correlated, it isn’t correlated. This paragraph is confusing because of you say positively or negatively correlated but there is no relationship. | This constructive critique, thanks, the statement has been checked and revised accordingly |
| I find it interesting that the trap counts and larval counts were correlated (except in Tanzania) – that relationship has been difficult to show in the Americas. | Yes, this is really an interesting finding that needs to be further investigated. |
| Fig. 8: how did you determine generations – based on larval size or on the calendar? It’s not based on moth counts and with a continuously breeding population I’m not sure where one generation ends and the other begins since you must have found a lot in between. | Thank you for the concern. We estimated FAW lifecycle duration to 1 month and a regular maize cycle between 3-4 months. Hence by observing the curves, the maximum number of generations which could be expected from an endogenous population from seedling to maturity is 3. However, we concur with the reviewer’s explanation. Invasions are random and factors such as temperature and host plant availability as well as crop stage might influence population dynamic. We added a table which shows both mean number of adult and larval stages in the six countries at different crop stages. |
| line 346 and Figs. 10 & 11: how are you measuring FAW density? I don’t see the data for trap captures vs. larval numbers in these figures. Also, why is there a constant population with two foci in western Ethiopia, even when it isn’t maize season? Are there other crops there that maintain year long populations or is the climate different? | Thanks for the comment. The FAW density was calculated based on the number of points in a location, with a more substantial number of clustered points resulting in more substantial values. The two foci in western Ethiopia coincide with irrigation schemes along the lake Tana river and lakes in the Rift valley where crops such as maize are maintained year long. This analysis helps in identifying the hot-spot areas with high infestation rates. |
| line 380: even though the push-pull system has potential, at only < 1% of the acreage I’m not sure how you can do any statistical analysis that is appropriate | Analysis has been redone. In this context the reviewer has a point and the overall appreciation of the results has been taken into consideration. |
| lines 437 – 440: I’m surprised you didn’t mention Varella et al. 2015, whose life-table studies document the effects of abiotic mortality (doi:10.1371/journal.pone.0130437). | We thank the reviewer for this valuable reference. This has been integrated in the discussion. |
| line 471: where is the reference for this? Species in this genus are known to attack whiteflies and relatively recently Tuta in tomato. Will it even survive in maize? Do you have a citation? I don't know of any mirids that attack moth adults and especially pupae (do they go underground?)? | Thank you for pointing this out. This was from a study that conducted by a team from the University Cheikh Anta Diop from Dakar and presented at the African Association of Insect Scientists AAIS November 2019, Abidjan. |
| lines 473 – 479: these lines are not necessary as you did not of this kind of work | We accept the reviewer’s suggestion and deleted the lines. |
| Discussion: Throughout the discussion you talk about the use of synthetic insecticides and the lack of the use of other approaches such as push-pull, bioinsecticides, or biological control. This is good documentation but give no reason for why the other approaches aren’t used. Do you have any information on the availability or cost of these other methods? As you know if almost always comes down to economics. | We thank the reviewer for this remark. Currently FAW is still a new pest in Africa and many development organizations are still developing strategies for its management. In the meantime, chemical pesticides are predominantly promoted by both governments and private sector. Other approaches are still under experimental stage for proof of concept. Once proven they will require a scaling strategy for large scale adoption. This will also depend on their affordability.  We have added statement on line 449-455 as suggested. |
