# Supplementary material for: Bioecology of fall armyworm Spodoptera frugiperda (J. E. Smith), its management and potential patterns of seasonal spread in Africa
Source: PLoS One. 2021 Jun 11;16(6):e0249042. doi: 10.1371/journal.pone.0249042 (PMC8195398; doi:10.1371/journal.pone.0249042)
Supplement: Attachment — Submitted filename: PONE-D-20-10745_2020_ver2.docx [file pone.0249042.s003.docx]

PONE-D-20-10745

Bioecology of fall armyworm *Spodoptera frugiperda* (J. E. Smith), its management and potential migration pattern in Africa; S. Niassy

This is my second review of this manuscript and the authors did a nice job making some of the corrections requested by the reviewers. However, there are still some issues that need to be processed. One important issue is a frame of reference on the relationship between cropping systems, rotation crops, and FAW populations. For example, in the abstract, lines 36-37, you have results that showed higher FAW populations in farms that practice intercropping and crop rotation, but you can’t say that intercropping and crop rotation increased FAW infestation. I know it’s a subtle difference, but the first statement describes the results that were found, your description is a cause and effect tone that I don’t think is justified.

Other comments:

line 83: northern rather than Northern

line 91: …eastern African countries (Burundi, Ethiopia…

line 109: …in East Africa for a better…

line 118: …locations for larval scouting and trapping of FAW males…

This brings up another point. I’ve always used larval as an adjective before the nouns sampling, or scouting, etc. rather than use the nouns larva or larvae. I believe it is still correct, but the editors will have a better idea.

lines 133-137: you say the same app was used but were the same methods used – same or similar traps, similar pheromone lures, time between sample collection, number of farms sampled, etc.

line 142: Traps and lures (unknown pheromone blend) were… You mention the company but that doesn’t give anyone an idea of what the blend was – how many pheromone components or their percentages. Unless you have done the chemical analysis to determine either what is added or what is released, you have to say the blend is unknown. Most pheromone companies will not give you that information because it is proprietary (I’ve tried with that company and received a response that it contains a blend that is attractive to fall armyworm).

lines 162-163: *Desmodium* should be italicized

line 185: neonate rather than neonates

line 222: were rather than was; larval rather than larva

line 228 and 236: the question of the difference between 4165 and 5291 was brought up by another reviewer and your response was that those were numbers of observations. Our question is why are the numbers different and how were they calculated? Both numbers relate to the targeted districts so they should be the same. I must be missing something.

line 232: this sentence should read something like, FAW trap counts were significantly lower in farms where the main crops were cowpeas, fruit trees, other grasses, vegetables, rice, and sorghum, relative to maize.

line 239: intercrops rather than intercrop

lines 245-247: this sentence should read something like, Higher numbers of moths were found under intercropping (2x) and crop rotation systems (3x) relative to the seasonal cropping system (Table 2). Moth numbers from the push-pull technology system were similar to those from the seasonal cropping system. In Table 2 you have push-pull as NS.

lines 249-251: according to your graph, Burundi does not have maize, root and tuber crops not in Uganda, and cassava is in Burundi. Therefore, these sentences should read, …practice in Rwanda and Tanzania. Crop rotation with millet was widely practiced in Ethiopia, root and tuber crops were mainly used as rotation crops in Burundi, while cassava/manioc was used in Uganda (Figure 5).

line 253: remove cassava and peanut as they were both NS

line 266: replace between with among

lines 266-272: where is the data for these statements? Also, you mention traps counts – are these number of moths per trap averaged for the area? Larval counts – number of larvae per plant or number of plants infested with larvae? If it is the latter, then perhaps say percent infested plants rather than larval counts – number of larvae per 50 plants as a percentage. So then did you have these percentages per farm and averaged per area?

lines 273-278: what brands of biopesticide where used? There were all commercialized products so should have a brand name and company. *thuringiensis* rather than thurengiensis

line 291: …coinciding with a low rainfall…

line 293: should be written, In Uganda, very low numbers of adult moths were found and there was one peak of larval infestation noted from September (Figure 7C). In Rwanda and Burundi, the peak increase in FAW abundance started between November and December (Figure 7D,E), and although larval abundance declined in both countries, moth numbers increased in Rwanda (Figure 7E).

line 304: crop rather than corp

lines 304-309: please replace with, A country by country analysis for FAW moth counts occurring at different maize crop phenologies suggested significant differences among stages in all countries except Rwanda, which had similar numbers for all stages (Table 4A). Most countries recorded high moth numbers for the vegetative and reproductive stages, although numbers were relatively low in Ethiopia and Tanzania for all stages. Uganda had high numbers of moths during the sowing stage and then low number for the rest of the crop cycle. For infested plants, all stages produced similar percentages in Kenya and Rwanda (Table 4B). Lower numbers of infested plants were found during the maturity stage in Burundi, Ethiopia, and Uganda; Tanzania had the lowest percent infested plants at the seeding stage.

The means in Table 4 don’t add correctly – for example under sowing for adult count, the mean I get is 9.1 not 5.3 and under seedling I get 10.0 not 8.0, so I’m not sure how these were calculated.

What happened to Figure 8? If you chose to use it, please explain what the bars represent.

Section 3.5: This is a really interesting part of the paper but I have two main points. Number 1: since many of your readers may not be from Africa, you need to have a map with the countries and lakes identified. Fig. 1 is good for the east Africa sampling sites, but since you talk about many other countries and four large lakes, your readers need to have a frame of reference. Number 2: as in other parts of the paper, what are your values? Fig. 9 is labeled trap counts, but what do these numbers represent – moths per trap per region, total number of moth collected, moths per site totaled over the sampling time, etc. It is not clear what is being displayed. Was the data from all other countries sampled the same way – I think I brought this up before. Figs. 10 and 11 are labeled FAW density – density of what, moths, larvae? This is unclear and the reader has no idea what the ranges are for each of the categories.

line 341: …trap counts, including sorghum, Africa’s…

line 379: citation 10 is not correct for this statement

lines 384-386: although this statement is most likely correct, you don’t provide any research to back it up. I suggest after infestation in 386, Although the negative effects of insecticides haven’t been tested against the natural enemies identified in Africa, studies from the Americas have shown these effects in related parasitoid and predator species (Souza et al. 2013; Impact of insecticides used to control *Spodoptera frugiperda* (J.E. Smith) in corn on survival, sex ratio, and reproduction of *Trichogramma pretiosum* Riley offspring; Chilean Journal of Agricultural Research 73: 122-127; Badji et al. 2004; Impact of deltamethrin on arthropods in maize under conventional and no-tillage cultivation; Crop Protection 23: 1031-1039).

line 399: rephrase, The study also noted that on farms where biopesticides were used, percent infested plants was higher.

lines 402-404: add another citation, Generally biopesticides are less toxic and sustainable, although they may negatively influence natural enemy populations (Penagos et al. 2005; Lethal and sublethal effects of the naturally derived insecticide Spinosad on parasitoids of *Spodoptera frugiperda* (Lepidoptera: Noctuidae); Biocontrol Science and Technology 15: 81-95). However, biopesticide use needs to be…

lines 417-420: remove sentence starting with Wakisaka et al. as DBM and FAW are not very similar.

line 420: …rainfall and the FAW…

line 421: coincided rather than coincides

line 422: followed rather than follows

line 423: coincided rather than coincides

line 430: …FAW density was significantly… and …whereas plant infestation was mainly influenced by the reproductive…

line 439: put available after strategies

lines 443-444: remove the phrase, …removal of adult males through pheromone-baited traps and… Therefore, just include initial release of parasitoids, hand picking, and egg crushing as techniques. Removal of males with pheromone traps has not been tested as a management strategy, as far as I know, with field crop species.

line 448: mirids can be important predators of caterpillars but I’ve not heard of adult moth predation. Like the other reviewer mentioned the first time, since it is only a personal communication, I would not include it here. Let K. Diarra publish the work and then it can be used.

line 449: remove “the” before push-pull

line 451: …companion plants that are currently imported from… and italicize Desmodium and Brachiaria

line 452: …would bring the cost of…

line 455: …farmers at no cost, although with little known efficacy.

line 458: include citations 12 and 26 after first sentence

line 467: remove “on”

line 468: space after winter

line 469: as the authors know, there has been a lot of research conducted with *S. exempta*, which is also a migrant. Therefore, I would add after (51), similar to *S. exempta* (Dewhurst et al. 2001; The relationship between outbreaks, rainfall and low density populations of the African armyworm, *Spodoptera exempta*, in Kenya; Entomologia Experimentalis et Applicata 98: 285-294; and Lee et al. 2004; A comparison of nutrient regulation between solitarious and gregarious phases of the specialist caterpillar, *Spodoptera exempta* (Walker); Journal of Insect Physiology 50: 1171-1180).

line 483: …pesticide applications, save costs for farmers, and…

References: this section needs to be reworked for consistency. The authors mentioned using a reference software (EndNote?). If so, they need to make sure that the citations are inserted into EndNote in the same fashion – sentence case, scientific names italicized, journals with the accepted abbreviations, etc.

Table 3: “beans” if you want to be consistent with Tables 1 and 2

Table 4: remove (scouted larva); …FAW larval-infested plants; I already mentioned that the means don’t seem to be correct

Figure legends: Figs. 10 and 11 need to have the variables identified – what are fall armyworm dynamics – adults, infested plants, both?

Fig. 12 needs much more clarification (also quarterly misspelled). What density are we talking about? Even as a tif file it is hard to understand, and the potential migration pattern(s) isn’t obvious. For example, in western Ethiopia you have an intersect with apparently high density and “permanent” populations. If so, where do they go – north and east within Ethiopia or south to Kenya? It is an interesting map but I’m not sure what it says other than most of east Africa from Ethiopia south to northern South Africa is highly infested, as is west Africa in the Ghana, Togo, and Benin area and countries around Côte d’Ivoire.
