# Supplementary material for: Bioecology of fall armyworm Spodoptera frugiperda (J. E. Smith), its management and potential patterns of seasonal spread in Africa
Source: PLoS One. 2021 Jun 11;16(6):e0249042. doi: 10.1371/journal.pone.0249042 (PMC8195398; doi:10.1371/journal.pone.0249042)
Supplement: Attachment — Submitted filename: Response to reviewers comments_final_22-01-2021.docx [file pone.0249042.s004.docx]

**Responses to Reviewers’ Comments**

**Manuscript ID: PONE-D-20-31046**

**Manuscript** title: Bioecology of fall armyworm *Spodoptera frugiperda* (J. E. Smith), its management and potential migration pattern in Africa

**Reviewers Comments to the Author**

Please use the space provided to explain your answers to the questions above. You may also include additional comments for the author, including concerns about dual publication, research ethics, or publication ethics. (Please upload your review as an attachment if it exceeds 20,000 characters)

The authors would like to thank the reviewers and editor for their valuable and pertinent comments and suggestions to improve the revised version of our manuscript. The point-by-point responses to the reviewers' comments and suggestions are listed as follows:

| **Reviewer #1**: |  |
| --- | --- |
| My comments are on an attached Word document.  This manuscript provides valuable information for farmers, extension workers, and scientists in east Africa related to fall armyworm. It will give baseline information for future research.  This is my second review of this manuscript and the authors did a nice job making some of the corrections requested by the reviewers. However, there are still some issues that need to be processed. One important issue is a frame of reference on the relationship between cropping systems, rotation crops, and FAW populations. For example, in the abstract, lines 36-37, you have results that showed higher FAW populations in farms that practice intercropping and crop rotation, but you can’t say that intercropping and crop rotation increased FAW infestation. I know it’s a subtle difference, but the first statement describes the results that were found, your description is a cause and effect tone that I don’t think is justified. | IRR is a relative risk measure and we have modified the explanation to reflect this meaning. |
| ***Other comments:*** |  |
| line 83: northern rather than Northern | Done |
| line 91: …eastern African countries (Burundi, Ethiopia… | Done |
| line 109: …in East Africa for a better… | Done |
| line 118: …locations for larval scouting and trapping of FAW males…  This brings up another point. I’ve always used larval as an adjective before the nouns sampling, or scouting, etc. rather than use the nouns larva or larvae. I believe it is still correct, but the editors will have a better idea. | Done |
| lines 133-137: you say the same app was used but were the same methods used – same or similar traps, similar pheromone lures, time between sample collection, number of farms sampled, etc. | We thank the reviewer for the comment, we have clarified the statement. |
| line 142: Traps and lures (unknown pheromone blend) were… You mention the company but that doesn’t give anyone an idea of what the blend was – how many pheromone components or their percentages. Unless you have done the chemical analysis to determine either what is added or what is released, you have to say the blend is unknown. Most pheromone companies will not give you that information because it is proprietary (I’ve tried with that company and received a response that it contains a blend that is attractive to fall armyworm). | We thank the reviewer for the suggestions. The pheromone blend consisted in four compounds (Z9-14Ac (81.7%); z11-16Ac (17.54%); Z7-12Ac (0.5%) and z9-12Ac (0.25%)). We have added the details as suggested. |
| lines 162-163: *Desmodium* should be italicized | Done |
| line 185: neonate rather than neonates | Done |
| line 222: were rather than was; larval rather than larva | Done |
| line 228 and 236: the question of the difference between 4165 and 5291 was brought up by another reviewer and your response was that those were numbers of observations. Our question is why are the numbers different and how were they calculated? Both numbers relate to the targeted districts so they should be the same. I must be missing something. | Thank you for your remark. However, these figures cannot be the same. N= 4165 represents number of farmers cultivating Maize and Sorghum as major crops. Meaning there are some other farmers cultivation other crop as main crop. N=5291 gave the number of farmers practicing seasonal and rotation cropping. Others do other cropping systems. |
| line 232: this sentence should read something like, FAW trap counts were significantly lower in farms where the main crops were cowpeas, fruit trees, other grasses, vegetables, rice, and sorghum, relative to maize. | We thank the reviewer for these constructive comments. The statement has been changed as suggested. |
| line 239: intercrops rather than intercrop | Done |
| lines 245-247: this sentence should read something like, Higher numbers of moths were found under intercropping (2x) and crop rotation systems (3x) relative to the seasonal cropping system (Table 2). Moth numbers from the push-pull technology system were similar to those from the seasonal cropping system. In Table 2 you have push-pull as NS. | Done |
| lines 249-251: according to your graph, Burundi does not have maize, root and tuber crops not in Uganda, and cassava is in Burundi. Therefore, these sentences should read, …practice in Rwanda and Tanzania. Crop rotation with millet was widely practiced in Ethiopia, root and tuber crops were mainly used as rotation crops in Burundi, while cassava/manioc was used in Uganda (Figure 5). | Done |
| line 253: remove cassava and peanut as they were both NS | Done |
| line 266: replace between with among | Done |
| lines 266-272: where is the data for these statements? Also, you mention traps counts – are these number of moths per trap averaged for the area? Larval counts – number of larvae per plant or number of plants infested with larvae? If it is the latter, then perhaps say percent infested plants rather than larval counts – number of larvae per 50 plants as a percentage. So then did you have these percentages per farm and averaged per area? | The data is available. We developed pivot tables which highlighted the various chemical and biopesticides used in each country as well as their quantities represented in Figure 6. Trap counts represent the number of moths in each trap while the larval count represent the number of plants infested with larvae. |
| lines 273-278: what brands of biopesticide where used? There were all commercialized products so should have a brand name and company. *thuringiensis* rather than thurengiensis | On the FAMEWS App, only the active agent e.g., Beauveria, Metarhizium, Bt or NPV were given as options. Therefore, we did not have a reliable access of the brand. The names of the biopesticides listed in line 273-277 are what appeared in the dataset that we used to make the analysis. |
| line 291: …coinciding with a low rainfall… | Done |
| line 293: should be written, In Uganda, very low numbers of adult moths were found and there was one peak of larval infestation noted from September (Figure 7C). In Rwanda and Burundi, the peak increase in FAW abundance started between November and December (Figure 7D,E), and although larval abundance declined in both countries, moth numbers increased in Rwanda (Figure 7E). | We thank again the reviewer for these constructive comments. The statement has been changed as suggested. |
| line 304: crop rather than corp | Done |
| lines 304-309: please replace with, A country by country analysis for FAW moth counts occurring at different maize crop phenologies suggested significant differences among stages in all countries except Rwanda, which had similar numbers for all stages (Table 4A). Most countries recorded high moth numbers for the vegetative and reproductive stages, although numbers were relatively low in Ethiopia and Tanzania for all stages. Uganda had high numbers of moths during the sowing stage and then low number for the rest of the crop cycle. For infested plants, all stages produced similar percentages in Kenya and Rwanda (Table 4B). Lower numbers of infested plants were found during the maturity stage in Burundi, Ethiopia, and Uganda; Tanzania had the lowest percent infested plants at the seeding stage. | We thank the reviewer for these constructive comments. The statement has been changed as suggested. |
| The means in Table 4 don’t add correctly – for example under sowing for adult count, the mean I get is 9.1 not 5.3 and under seedling I get 10.0 not 8.0, so I’m not sure how these were calculated. | The means were calculated using pivot table in Excel. The table means are correct. The number of observations for stage for a country were not the same. So, calculating the marginal means using the ‘inside means’ is not correct because you give the means equal weighting (sum of mean values/6) which is not appropriate. The displayed means are therefore correct and are retained - no change. You would get correct values if n was the same for each cell. |
| What happened to Figure 8? If you chose to use it, please explain what the bars represent. | We reinstated the paragraph that explained figure 8 in section 3.4 – line 305 - 311. I further made reference to the colour bars of each of the phenology stages. |
| Section 3.5: This is a really interesting part of the paper but I have two main points.  Number 1: since many of your readers may not be from Africa, you need to have a map with the countries and lakes identified. Fig. 1 is good for the east Africa sampling sites, but since you talk about many other countries and four large lakes, your readers need to have a frame of reference.  Number 2: as in other parts of the paper, what are your values? Fig. 9 is labeled trap counts, but what do these numbers represent – moths per trap per region, total number of moth collected, moths per site totaled over the sampling time, etc. It is not clear what is being displayed. Was the data from all other countries sampled the same way – I think I brought this up before. Figs. 10 and 11 are labeled FAW density – density of what, moths, larvae? This is unclear and the reader has no idea what the ranges are for each of the categories. | We added explanation to Fig 9 in the text, line 323-324. Figures in the legend represent the summation of all the adults and larvae collected quarterly.  We updated captions for Fig 10 and 11. The maps represent FAW adults counts and this has been captured in the text in line 349.  Fig 12 map was improved by adding lakes as well as the labels for the countries for easier reference. Values were categorized into five classes: very low, low, moderate, high and very high for easier interpretation. |
| line 341: …trap counts, including sorghum, Africa’s… | Done |
| line 379: citation 10 is not correct for this statement | Done |
| lines 384-386: although this statement is most likely correct, you don’t provide any research to back it up. I suggest after infestation in 386, Although the negative effects of insecticides haven’t been tested against the natural enemies identified in Africa, studies from the Americas have shown these effects in related parasitoid and predator species (Souza et al. 2013; Impact of insecticides used to control *Spodoptera frugiperda* (J.E. Smith) in corn on survival, sex ratio, and reproduction of *Trichogramma pretiosum* Riley offspring; Chilean Journal of Agricultural Research 73: 122-127; Badji et al. 2004; Impact of deltamethrin on arthropods in maize under conventional and no-tillage cultivation; Crop Protection 23: 1031-1039). | Done |
| line 399: rephrase, The study also noted that on farms where biopesticides were used, percent infested plants was higher. | Done |
| lines 402-404: add another citation, Generally biopesticides are less toxic and sustainable, although they may negatively influence natural enemy populations (Penagos et al. 2005; Lethal and sublethal effects of the naturally derived insecticide Spinosad on parasitoids of *Spodoptera frugiperda* (Lepidoptera: Noctuidae); Biocontrol Science and Technology 15: 81-95). However, biopesticide use needs to be… | Done |
| lines 417-420: remove sentence starting with Wakisaka et al. as DBM and FAW are not very similar. | We thank the reviewer for this comment. This statement illustrates the relationship between rainfall and pest infestation. Although DBM and FAW are not similar, they are both lepidopteran pests. |
| line 420: …rainfall and the FAW… | Done |
| line 421: coincided rather than coincides | Done |
| line 422: followed rather than follows | Done |
| line 423: coincided rather than coincides | Done |
| line 430: …FAW density was significantly… and …whereas plant infestation was mainly influenced by the reproductive… | Done |
| line 439: put available after strategies | Done |
| lines 443-444: remove the phrase, …removal of adult males through pheromone-baited traps and… Therefore, just include initial release of parasitoids, hand picking, and egg crushing as techniques. Removal of males with pheromone traps has not been tested as a management strategy, as far as I know, with field crop species. | Done |
| line 448: mirids can be important predators of caterpillars but I’ve not heard of adult moth predation. Like the other reviewer mentioned the first time, since it is only a personal communication, I would not include it here. Let K. Diarra publish the work and then it can be used. | Done |
| line 449: remove “the” before push-pull | Done |
| line 451: …companion plants that are currently imported from… and italicize Desmodium and Brachiaria | Done |
| line 452: …would bring the cost of… | Done |
| line 455: …farmers at no cost, although with little known efficacy. | Done |
| line 458: include citations 12 and 26 after first sentence | Done |
| line 467: remove “on” | Done |
| line 468: space after winter | Done |
| line 469: as the authors know, there has been a lot of research conducted with *S. exempta*, which is also a migrant. Therefore, I would add after (51), similar to *S. exempta* (Dewhurst et al. 2001; The relationship between outbreaks, rainfall and low density populations of the African armyworm, *Spodoptera exempta*, in Kenya; Entomologia Experimentalis et Applicata 98: 285-294; and Lee et al. 2004; A comparison of nutrient regulation between solitarious and gregarious phases of the specialist caterpillar, *Spodoptera exempta* (Walker); Journal of Insect Physiology 50: 1171-1180). | Done |
| line 483: …pesticide applications, save costs for farmers, and… | Done |
| References: this section needs to be reworked for consistency. The authors mentioned using a reference software (EndNote?). If so, they need to make sure that the citations are inserted into EndNote in the same fashion – sentence case, scientific names italicized, journals with the accepted abbreviations, etc. | Done |
| Table 3: “beans” if you want to be consistent with Tables 1 and 2 | Done |
| Table 4: remove (scouted larva); …FAW larval-infested plants; I already mentioned that the means don’t seem to be correct | Done |
| Figure legends: Figs. 10 and 11 need to have the variables identified – what are fall armyworm dynamics – adults, infested plants, both? | The maps represent FAW adults across the maize seasonality dynamics. This has been updated on the text, line 343-347; the data was for adults. |
| Fig. 12 needs much more clarification (also quarterly misspelled). What density are we talking about? Even as a tif file it is hard to understand, and the potential migration pattern(s) isn’t obvious. For example, in western Ethiopia you have an intersect with apparently high density and “permanent” populations. If so, where do they go – north and east within Ethiopia or south to Kenya? It is an interesting map but I’m not sure what it says other than most of east Africa from Ethiopia south to northern South Africa is highly infested, as is west Africa in the Ghana, Togo, and Benin area and countries around Côte d’Ivoire. | We thank the reviewer for the comment. We have corrected the misspelling (see legend figure 12). We have also indicated potential migratory as well as lakes and rivers. The maize calendar varies within and between countries (See figure 7) and there is an alternation of seasons between regions. Most East African countries e.g., Kenya, Uganda, Rwanda, Ethiopia have two rainy seasons while southern African countries eg. Malawi, Zambia, Zimbabwe have one rainy season. Additionally, irrigation schemes around lakes and along rivers sustain FAW populations from where they expand to maize field during maize season. As migratory pest which prefers maize, the seasonality of the crop influences it migration pattern across regions.  These variations justify potential migratory routes with East Africa and between East and Southern Africa considering the long dry spell in southern African countries. In the case of Ethiopia, population might move northwards to Sudan following Nile river or Southwards. In West Africa, countries like Ghana and Cote d’Ivoire experience major and minor seasons. At the same time there are hot spots with irrigation schemes from which populations could invade other parts of the region. |
| **Reviewer #2:** |  |
| This paper documents a monitoring and data collection system via a mobile app across a wide geographic area, spanning multiple countries, a remarkable achievement for IPM. The effort speaks to a technological and sociological effort that appears to be sustainable and adaptable to other species. I think the authors miss an opportunity to emphasize this in the discussion.  The authors mine a rich dataset from this system to describe the current population dynamics of an invasive species that threatens food security and test for variables that influence these dynamics. There is a pressing need to get this information out to a wide audience, and I think this paper should be published. The dynamics of invasive species may well change, thus the dataset has historical value.  The language about testing the influence of explanatory variables with a negative binomial (NB) model is confusing. My understanding is that a NB model is used to fit a probability density function, or a frequency distribution, and I understand why this would be a good choice when working with count data. However, a NB model does not test for significance of explanatory variables. For example, Tables 1-3, report regression estimates and their significance. From reading the responses to previous reviews, it says the authors used “…generalized linear model that assume a negative binomial distribution error …. This model uses the MASS package which has been included in the ‘Data analysis section’ “This is useful – but it is NOT in the data analysis section!  Briefly explain what IRR refers to in the section on statistical methods, and how to interpret this statistic. Also, clarify that you are developing this metric for the data across the entire time frame of this dataset (at least, that’s what I think you are doing, right?) | Just like ANOVA tests the effects of ‘treatments’ using f-test, Negative Binomial model tests the effect of “treatments”/” explanatory variables” using Likelihood ratio Chi-square (also known as deviance or to be more precise ‘change in deviance from null model”). Since NB model is a regression model, the regression estimates must be interpreted, while in ANOVA, the treatment means under test, must be explained.  The fitting of NB model has the same format as the fitting of ANOVA.  ANOVA fitting in R:  **aov**(response~treatment, data= data1)  NB model fitting in R  **glm.nb**(response~treatment, …, data= data1)  We have clarified on the term ‘IRR’ and its interpretation as recommended:  IRR is a relative measure of incidence rate, thus a ratio of two incidence rates - and so one should be a base rate (e.g. exposed group vs unexposed group)  IRR = 1 means no effect of the exposure  IRR > 1 means positive effect of exposure  IRR < 1 means negative effect of exposure |
| What are the sample sizes for the analyses in Tables 1-3. Please give the degrees-of-freedom for the regression estimates in Tables 1-3. I am trying to figure out if I am looking at tests using thousands of datapoints, or if the data were organized into means (per site, month, whatever?) prior to running the regression. | We have indicated the sample sizes for analysis for Tables 1-3 as follows:  Table 1 Main crop n = 3438  Table 2 Cropping system n = 3423  Table 3 Rotation crop n = 1479 |
| Lines 232-234 – why did you not include beans since it also has a significant regression estimate (albeit at only one *). | Done |
| Lines 246-247: you claim push-pull reduces infestation relative to seasonal, but Table 2 shows a ns regression estimate and an IRR of almost 1. If I am reading Table 2 correctly, you were NOT able to discern a significant effect from push-pull. The 95% CI for the IRR, however, is very large. Perhaps the conclusion is that you see wide variation in the effect of push-pull, perhaps due to a small sample size? | Done |
| Line 252-253 – why are you including cassava and peanut, when Table 3 shows a ns regression estimate? | Done |
| Please report the units for the variables of trap count and do this throughout the manuscript and in the tables and figures. Moths/trap/week? Mean of moths/trap/week across some spatial area? Means of moth/trap/month (which I think is the case for figure 7?) What about Figure 8? Readers need to understand the time component of the unit (week? month? entire study dataset?) for each analysis / figure / table. I found this text in the response to earlier reviews: “Trap counts (adult counts) is the total number of moths in traps. Scouting count refers to the number of infested plants recorded in a sample of 50 plants per field.” This is helpful – but the analyses/tables/figures lead me to believe that some of the time I am looking at means or sums – this needs to be clarified. | We used the sum of all the observation records across the study period in all the countries. For figure 8, the numbers in the y-axes represent the summation of all the observations for the entire study period, across the six countries. As for figures 7, the total adults and larvae counts are indicated for the individual countries. |
| The same comment for the variable ‘scouting data’ – what units we are looking at? Are the data being summed, or averaged, over some unit of space or time? It seems to vary at different parts of the text and figures/tables. | The figures represent the sum of the observation counts. |
| Figure 8 is not mentioned in the text. Also, what are the units in the y-axis of Figure 8 referring to? They are in values of thousands, and the axis is labelled as ‘crop stage’. What does that refer to? | Both y-axes have labels, the y-axis on the left is labelled “number of FAW” representing the trap counts while the y-axis on the right is labelled “number of plants” representing the larval counts. The x-axis is labelled “crop stage” representing the different phenology stages of the crop. |
| The terms ‘seasonal cropping’ and ‘crop rotation’ are being used in a way that is different than other parts of the world. To clarify, include the explanation that you placed in the response to reviewers in the intro (Seasonal cropping is a farming practice in which the same crop is grown in the same area for a number of growing seasons. In the case of maize, it is mainly rain fed, and the land remain fallow between seasons. While crop rotation is the practice of growing a series of different types of crops in the same area across seasons.) | Done |
| For the UniTraps buckets – did you use the all green version, or the yellow/white/green version, or was this unknown? | We used the yellow version. |
| Lines 162-163 – give the Latin name for the Desmodium spp. and Napier grass, if possible. | More precision has been added |
| Line 190 – the word data is repeated | Done |
| Lines 341-342 – the sentence that starts with ‘Sorghum…” is not a sentence. | Done |
| Line 467 – remove ‘on’ | Done |
| Results section 3.3 – explain the terms ‘Meher’ and ‘Belg’ | Done |
| **Reviewer #3:** |  |
| This manuscript is much improved over earlier iterations, and I believe it is suitable for publication from my point of view after considering the comments below. My major concern is still the following, even though the authors have partially addressed the problem: | We sincerely thank the reviewer for his suggestions on the manuscript. |
| L204-207: The methodology described does not and cannot “determine FAW migratory patterns at the regional level”, nor can it “indicate the movement of the adult moths across the region.” The trap counts and heat maps are generated from sampling data and only show the change in FAW density over time in each location, and differences in FAW density between locations at a point in time. These data by themselves cannot distinguish whether density changes over time and density differences across space are caused by migration or by population growth/decline from local reproduction and mortality. One or the other or a combination of both could be occurring. Some of the responses to past reviews at first made me think the authors understand this in principle, but sentences like these suggest maybe not. It is known that FAW is a migratory species in North America. Everybody knows this is a migratory species. But no one knows yet how that migratory behavior (which is complicated enough in the Americas) translates to Africa or Asia. Given the rate of spread, FAW is surely exhibiting migratory behavior in its new hemisphere, but what that looks like now in Africa is unknown. Demonstrating patterns and timing to and from different regions on the newly invaded continent of Africa will require additional kinds of data like radar, high-altitude sampling, back-track wind trajectory analyses, and so forth. Heat maps and pheromone trap captures can contribute to figuring out migratory patterns, mainly by generating hypotheses to test, but they simply cannot do so the way the authors seem to think. In my opinion, the authors must use more careful language; for example, they can say their data provide clues to possible migratory patterns, and form the basis of hypotheses about movement patterns that can be tested. Generating meaningful hypotheses is not a small thing; good hypotheses are essential to making progress by not wasting time and resources on more speculative conjectures; the authors should emphasize this because this will be a major impact of their data. But they cannot say the heat maps and trap data “indicate the movement” of the moths or “determine migratory patterns” at any scale. It is possible the heat maps and trap data reflect such movement, but we cannot know this is true without additional, independent kinds of data. It will take a big effort (probably of multiple multidisciplinary teams over several years) of gathering and analyzing other kinds of data to eventually clarify patterns of FAW migratory movement in Africa. Because of these considerations, and because “migration” is a very specific yet complex kind of movement, I also suggest the authors change “potential migration pattern” in the title to “potential patterns of seasonal spread” (echoing verbiage on line 85), which is more defensible based on the data presented in this paper, and will be of equal interest to those concerned about FAW management. | As we clearly mentioned in the introduction and discussion, the Community Based Fall Armyworm Monitoring Forecasting Early Warning approach was meant to collect data and evidence of the presence or absence of FAW adults and larvae in a certain location at a certain time on a certain crop. This information if substantial could provide means the predict, model the pest dynamic across agroecologies. Based on the evidence-based nature of the research, the vast body of research on FAW in the Americas, and several parameters as highlighted in the materials we tried to explain FAW bioecology in Africa. While we acknowledge that more efforts are needed if we invested in this approach, information generated in this paper is not negligible because it also guides the success of any IPM effort towards controlling the pest.  The statements made in terms of main crop and cropping system effect, crop stage, rainfall effects are supported by previous research in the Americas and in Africa. We agree with the reviewers that this could be a potential migratory pattern and it has been suggested to include maps of waters on the continent.  We also assure the reviewer that more efforts using other approaches are being made using complementary data from the US and other places to define ecoclimatic index and other parameters such as altitude, heat, cold etc…  The current paper is a humble contribution in IPM research on FAW and can nevertheless serve as a baseline to better understand FAW in future. |
| ***Minor comments:*** |  |
| L35: change “Have implication on” to “impact” | Done |
| L50: change “crop” to “crops” | Done |
| L52: change “biotic constrants” to “biotic factors” | Done |
| L155: delete repeated instance of “and field scouting data” | Done |
| L190: delete first instance of “date” | Done |
| L209: I encourage the authors to use a different term than “intersect sites”, because it is not intuitive what this means, even though they define here how they will be using it in this paper. What the authors are describing is a region of year-round reproduction. Alternative, more intuitively descriptive terms for this could be: “permanent distribution” or “year-round distribution” or “area of year-round residency”. | Done |
| L213: “caters of overdispersion in integer counts data” This phrase is unclear. Suggest changing to “accommodates overdispersion of integer count data”. | Done |
| L219: delete first instance of “a” | Done |
| L232: delete hyphen from “Like-lihood” | Done |
| L302: change “larvae (scouting)” to “larval scouting” | Done |
| L326: change “built” to “build” | Done |
| L37, 332, 362: change “larvae” to “larval” | Done |
| L341-343: change to “Data suggest more attention is needed on other crops attacked by FAW such as sorghum (32), Africa’s second most important cereal in terms of tonnage (31). | Done |
| L466-468: “the possibility of a sporadic infestation can be speculated on in Africa compared to the Americas where FAW faces winter” The meaning of this whole phrase is unclear. What is meant by “sporadic infestation”? What does FAW “facing winter” in the Americas have to do with anything? The intended meaning and the logic are not clear. | We thank the reviewers for this observation. The term “sporadic” is replaced with the term “seasonal” in relation to the seasonal nature of maize cropping and the intra and inter variations of rainfall in the regions.  Most East African countries e.g., Kenya, Uganda, Rwanda, Ethiopia have two rainy seasons while southern African countries e.g., Malawi, Zambia, Zimbabwe have one rainy season. The maize calendar varies within and between countries and there is an alternation seasons between regions. These variations also justify potential migratory routes especially with the presence of irrigation schemes around lakes and along rivers which sustain FAW populations from where they expand to maize field during maize season.  This scenario, of course is different from that in North America where it is established that migration starts from Texas and Florida in the late winter or spring. We have indicated those migratory routes on Figure 12. |
| L488: delete “in” | Done |
| L677: change “Scounted” to “scouted” | Done |
