# Supplementary material for: Bioecology of fall armyworm Spodoptera frugiperda (J. E. Smith), its management and potential patterns of seasonal spread in Africa
Source: PLoS One. 2021 Jun 11;16(6):e0249042. doi: 10.1371/journal.pone.0249042 (PMC8195398; doi:10.1371/journal.pone.0249042)
Supplement: Attachment — Submitted filename: 2nd Response to reviewer.docx [file pone.0249042.s005.docx]

| Reviewer’s Comments | Responses |
| --- | --- |
| Reviewer #3: The authors have not addressed the main concern I voiced last time. I've pasted it again here, because they simply cannot say (Lines 209-213): "Further, data collected from pheromone traps (universal bucket traps) in the eastern and other African countries were quarterly mapped to determine FAW adults migratory patterns at the regional level. Specifically, we overlaid the quarter FAW density heat maps in 2018 and 2019 to indicate the movement of the adult moths across the region." Pheromone trap captures mapped in this way cannot indicate adult migratory patterns, and the density heat maps cannot indicate the movement of adult moths across the region. I explained previously why these assumptions are not supportable. I feel like we are talking past each other. | We thank the reviewer for this remark. We agree that heatmaps cannot determine FAW migratory pattern nor can they indicate FAW movement. We have therefore revised the statements in the main document.  We now understand the comments and will surely consider his suggestions on use of additional kinds of data like radar, high-altitude sampling, back-track wind trajectory analyses in subsequent studies. This has been highlighted in the revised manuscript. |
| Assuring me as the reviewer that "more efforts using other approaches are being made" misses the point entirely, because that does not make the assumptions underlying their statements on Lines 209-213 true. The assumptions remain incorrect. It also is not about the authors "making a humble contribution to IPM research on FAW"; I am happy they are making this contribution. The problem is that pheromone trap captures and mapping cannot "determine FAW adult migratory patterns." They simply cannot for the reasons I described previously (pasted here again): "The methodology described does not and cannot “determine FAW migratory patterns at the regional level”, nor can it “indicate the movement of the adult moths across the region.” The trap counts and heat maps are generated from sampling data and only show the change in FAW density over time in each location, and differences in FAW density between locations at a point in time. These data by themselves cannot distinguish whether density changes over time and density differences across space are caused by migration or by population growth/decline from local reproduction and mortality. One or the other or a combination of both could be occurring. Some of the responses to past reviews at first made me think the authors understand this in principle, but sentences like these suggest maybe not. It is known that FAW is a migratory species in North America. Everybody knows this is a migratory species. But no one knows yet how that migratory behavior (which is complicated enough in the Americas) translates to Africa or Asia. Given the rate of spread, FAW is surely exhibiting migratory behavior in its new hemisphere, but what that looks like now in Africa is unknown. Demonstrating patterns and timing to and from different regions on the newly invaded continent of Africa will require additional kinds of data like radar, high-altitude sampling, back-track wind trajectory analyses, and so forth. |  |
| Heat maps and pheromone trap captures can contribute to figuring out migratory patterns, mainly by generating hypotheses to test, but they simply cannot do so the way the authors seem to think. In my opinion, the authors must use more careful language; for example, they can say their data provide clues to possible migratory patterns, and form the basis of hypotheses about movement patterns that can be tested. | We really appreciate the reviewer’s comment. What was presented in this study is hypothetical and still need to be tested in future. Throughout the document, we used terms words such as “might”, “imply”, “possible” and “potential”. |
| Generating meaningful hypotheses is not a small thing; good hypotheses are essential to making progress by not wasting time and resources on more speculative conjectures; the authors should emphasize this because this will be a major impact of their data. But they cannot say the heat maps and trap data “indicate the movement” of the moths or “determine migratory patterns” at any scale. | Indeed, the reviewer is correct, and we have statements alluding to the same. |
| It is possible the heat maps and trap data reflect such movement, but we cannot know this is true without additional, independent kinds of data. It will take a big effort (probably of multiple multidisciplinary teams over several years) of gathering and analyzing other kinds of data to eventually clarify patterns of FAW migratory movement in Africa. | We appreciate the reviewer’s expertise in the field, and we will definitely consider his suggestion. One of the motives of the paper was indeed to sustain the efforts which led to the current study. It is our hope that the current results will trigger multidisciplinary teams to collect additional data and conduct more in-depth analysis to uncover FAW migratory pattern in Africa. This has been emphasized in the conclusion. |
| Because of these considerations, and because “migration” is a very specific yet complex kind of movement, I also suggest the authors change “potential migration pattern” in the title to “potential patterns of seasonal spread” (echoing verbiage on line 85), which is more defensible based on the data presented in this paper, and will be of equal interest to those concerned about FAW management." | Done |
| Why can the authors not change the wording so that they do not claim so much? What they claim in this regard is not defensible in my opinion. Not claiming that they now know the migration patterns in this part of Africa based on pheromone trap captures does not diminish the importance of their data. Instead, asserting that they now do know the migration patterns detracts from the data's importance by making such a mistake in interpretation. Changing the wording to say that the patterns they see in the pheromone trap captures and heat maps "suggest" that the FAW "may be" migrating in such-and-such a pattern in Africa would be fine. It is also fine to have the arrows in Fig. 12 indicating "Potential" migration routes, because this acknowledges that these potential routes are basically hypotheses to be tested, rather than conclusive findings regarding migration routes. | Point taken! Everything we stated regarding FAW migration in the paper was carefully conditional mode with words such as “imply” “potential” “possibilities” “might” see line 488-499. We do share the same opinion with the reviewer. |
| I would add that the sentence (L495-497) "FAW migratory scenario in Africa is different from that in North America where it is established that migration starts from Texas and Florida in the late winter or spring (49)" should be more carefully stated. This is because (1) of course it is different since Texas and Florida are not in Africa, making this a trivial/meaningless statement, and (2) they do not know what the migration pattern in Africa is. Their trapping and density data suggest hypotheses to test (such as in Fig. 12). I do not know what point the authors are trying to make by discussing Texas and Florida as the source for spring migrants to the north in North America, so they will need to clarify that point. Maybe something about differences in seasonality on the two continents? Differences in directionality because of North/South hemispheric differences in seasons? Based on the context of the sentence, when they say "migratory scenario" perhaps they mean to imply this is a "potential" scenario. If so (and I hope so), they need to say this explicitly, because this intent is not conveyed by the current language. | The statement regarding FAW migration in the America has been removed. |
| I don't know what else I am supposed to say. This is my best judgment. I hope they can make these simple but important changes in wording. The authors have addressed my other more minor concerns. | We thank the review for these critical comments which we believe improve the quality of the paper. |
